# Dihydrotanshinone I exhibits antitumor effects via β-catenin downregulation in papillary thyroid cancer cell lines

**DOI:** 10.1038/s41598-024-58495-9

**Published:** 2024-04-03

**Authors:** Elisabetta Molteni, Federica Baldan, Giuseppe Damante, Lorenzo Allegri

**Affiliations:** 1 Department of Medicine, University of Udine, 33100 Udine, Italy; 2 Institute of Medical Genetics, Academic Hospital of Udine, Azienda Sanitaria Universitaria Friuli Centrale, 33100 Udine, Italy

**Keywords:** Cancer, Transcriptomics, Cell growth, Cell migration

## Abstract

Thyroid cancer is the most common endocrine carcinoma and, among its different subtypes, the papillary subtype (PTC) is the most frequent. Generally, PTCs are well differentiated, but a minor percentage of PTCs are characterized by a worse prognosis and more aggressive behavior. Phytochemicals, naturally found in plant products, represent a heterogeneous group of bioactive compounds that can interfere with cell proliferation and the regulation of the cell cycle, taking part in multiple signaling pathways that are often disrupted in tumor initiation, proliferation, and progression. In this work, we focused on 15,16-dihydrotanshinone I (DHT), a tanshinone isolated from* Salvia miltiorrhiza* Bunge (Danshen). We first evaluated DHT biological effect on PTC cells regarding cell viability, colony formation ability, and migration capacity. All of these parameters were downregulated by DHT treatment. We then investigated gene expression changes after DHT treatment by performing RNA-seq. The analysis revealed that DHT significantly reduced the Wnt signaling pathway, which plays a role in various diseases, including cancer. Finally, we demonstrate that DHT treatment decreases protein levels of β-catenin, a final effector of canonical Wnt signaling pathway. Overall, our data suggest a possible use of this nutraceutical as an adjuvant in the treatment of aggressive papillary thyroid carcinoma.

## Introduction

Thyroid cancer (TC) is the most common endocrine carcinoma, accounting for 1–2% of cancer cases worldwide^1^. Most thyroid tumors arise from the epithelial cells of the gland and are classified into well-differentiated carcinoma (DTC), which includes papillary carcinoma (PTC, 80–85%), follicular carcinoma (FTC, 10–15%); poorly differentiated carcinoma (PDTC, 1–3%) and undifferentiated anaplastic thyroid carcinoma (ATC, < 2%) ^2–4^. Nowadays, the treatment of thyroid carcinoma includes surgery and radioiodine administration and is effective only for DTCs that are able to uptake radioiodine^5^. Nevertheless, these therapeutic approaches are not effective for PDTC and ATC^6^. In general, conventional papillary thyroid carcinoma is indolent with an associated 10-year survival greater than 95%^7,8^. However, 5–10% of PTCs shows a worse prognosis and a more aggressive behavior in the spectrum between well differentiated classic PTC and the undifferentiated anaplastic carcinoma, including higher rates of metastases, recurrence, resistance to radioactive iodine (RAI) therapy, and possibly compromised survival^9–12^. Knowledge of the molecular mechanisms that contribute to the aggressiveness of PTCs is critical to propose tailored therapy approaches.

The aim of this study is to evaluate the use of more effective and well-tolerated agents for thyroid cancer treatment, and, in particular, phytochemicals. Phytochemicals constitute a heterogeneous group of bioactive compounds classified by chemical structure and include polyphenols, alkaloids, carotenoids, and nitrogen compounds^13^. These compounds are naturally present in fruits, vegetables, grains, and other plant products and are often responsible for distinguished plant characteristics, such as color pigmentation and smell. Early studies indicated that these compounds are able to influence cell proliferation and cell cycle regulation, and usually participate in multiple signaling pathways that are often disrupted in tumor initiation, proliferation, and propagation^14–17^. Among the many phytochemicals tested in cancer cell lines, our study focused on 15,16-dihydrotanshinone I (DHT), a tanshinone extracted from Salvia miltiorrhiza Bunge (Danshen), one of the most frequently prescribed herbs in traditional Chinese medicine ^18^. A wide range of biological activities, such as, anti-platelet aggregation, anti-inflammatory, anti-tumor, and antibacterial activities against a broad range of gram positive bacteria, has been attributed to 15,16-dihydrotanshinone I ^19–22^. DHT has shown promising antitumor effects in numerous in vitro models of many types of cancer ^23–26^ and has also been tested in animal models, confirming its ability to inhibit tumor growth without adverse effects on healthy tissues^27,28^. Overall, these results indicate that DHT is a candidate as a new agent in cancer treatment. In this work, we investigate the biological effects of DHT as well as its biological mechanism of action on PTC cell lines.

## Results

### Effects of DHT on PTC cell viability and colony formation

**Figure 1:**
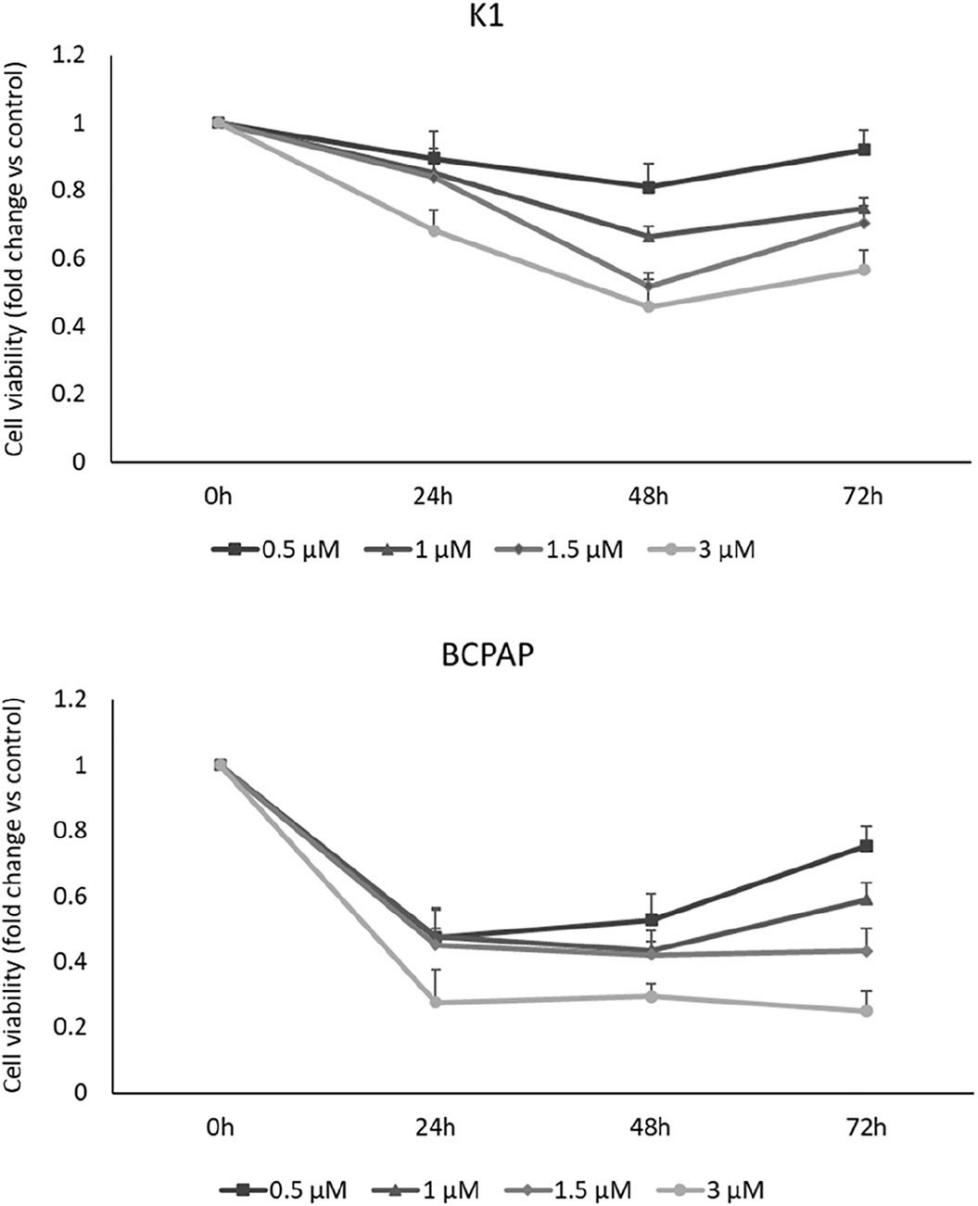
Effect of DHT on cell viability. K1 and BCPAP cells were treated with DHT at different doses (rising from 0.5 μM to 3 μM) or vehicle (DMSO) for 72 h and cell viability was assessed by MTT assay. Each point represents the mean of six measurements. n = 6. Statistical data are listed in the Supplementary Table 1.

In the first experimental setting, we evaluated the effect of DHT in two PTC cell lines, K1 and BCPAP, focusing on its effects on cell viability, colony formation and ability to migrate. As shown in Fig. 1, DHT was able to significantly reduce cell viability at different concentrations in both cell lines. Overall, BCPAPs seemed to be more sensitive to treatment, even at early times (24 h). Treatment with DHT 1.5 µM showed a reduction in cell viability greater than35% in both cell lines after 48 h of administration. Interestingly, the greatest impact compared with the control was observed at 48 h, while at 72 h no increase in DHT efficacy was detected, although cell viability was still markedly reduced compared to the control.

Using the data obtained from the MTT assay as input, the effective dose of DHT capable of reducing cell viability by 50% (ED50) was calculated. By the interpolation method, the average ED50 common to the two cell lines was calculated as 2.5 µM; that concentration was used for subsequent experiments.

**Figure 2:**
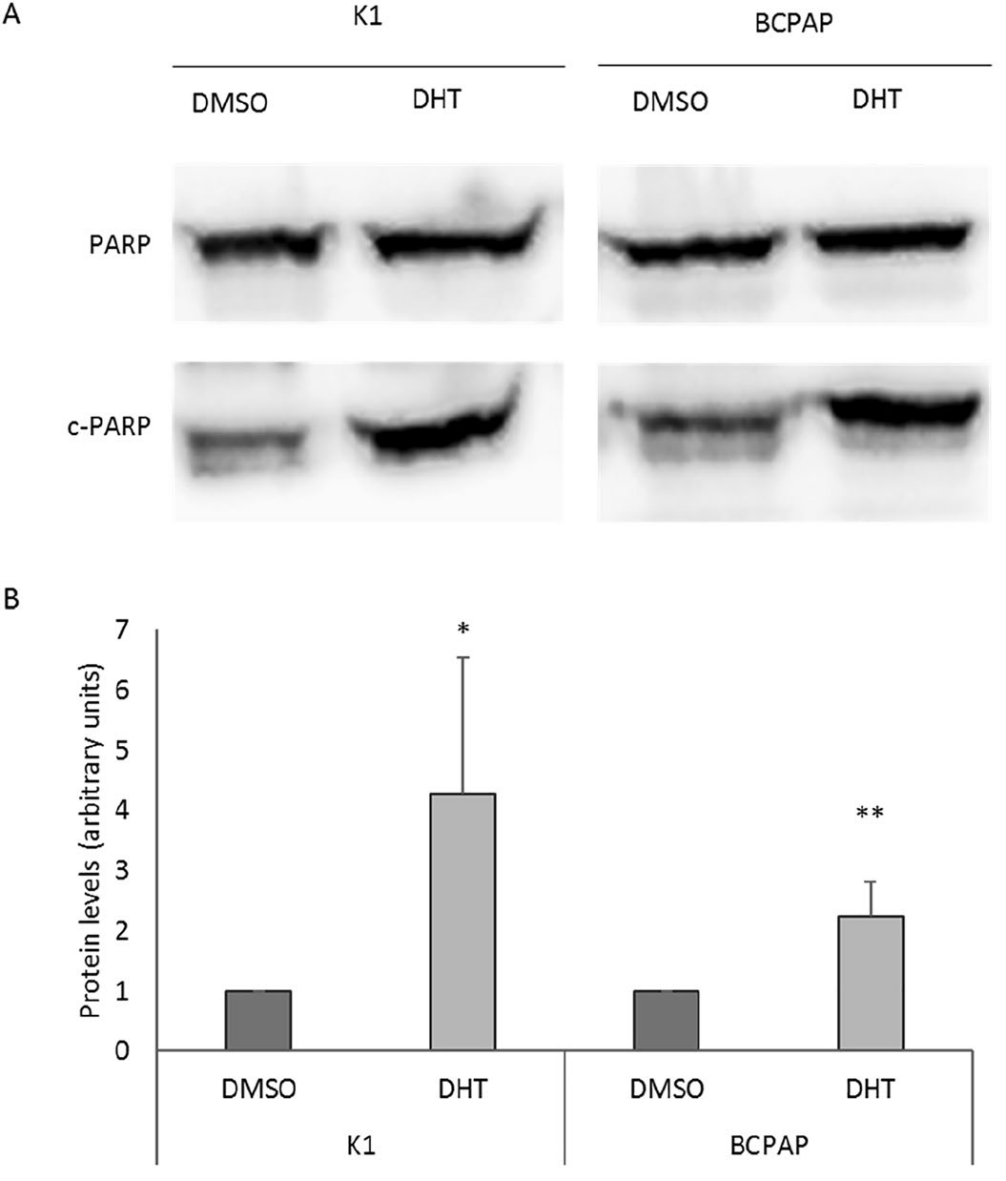
Effect of DHT on apoptosis. Cleaved-PARP (c-PARP) protein levels were evaluated as apoptosis marker by Western blot assay in K1 and BCPAP cells treated with vehicle (DMSO) or DHT 2.5 μM. For each cell line, the c-PARP levels were normalized against full-length PARP (PARP) levels and expressed as arbitrary unit. Data are representative of 3 independent experiments and results are shown as mean ± SD. *p < 0.05, **p < 0.01.

To assess whether the observed reduction of cellular viability after the treatments was attributable to apoptotic cell death, we evaluated the ratio between PARP and cleaved- PARP as marker of apoptosis (Fig. 2). As can be seen from the figure, a significant increase in apoptosis was observed in both cell lines following treatment.

**Figure 3:**
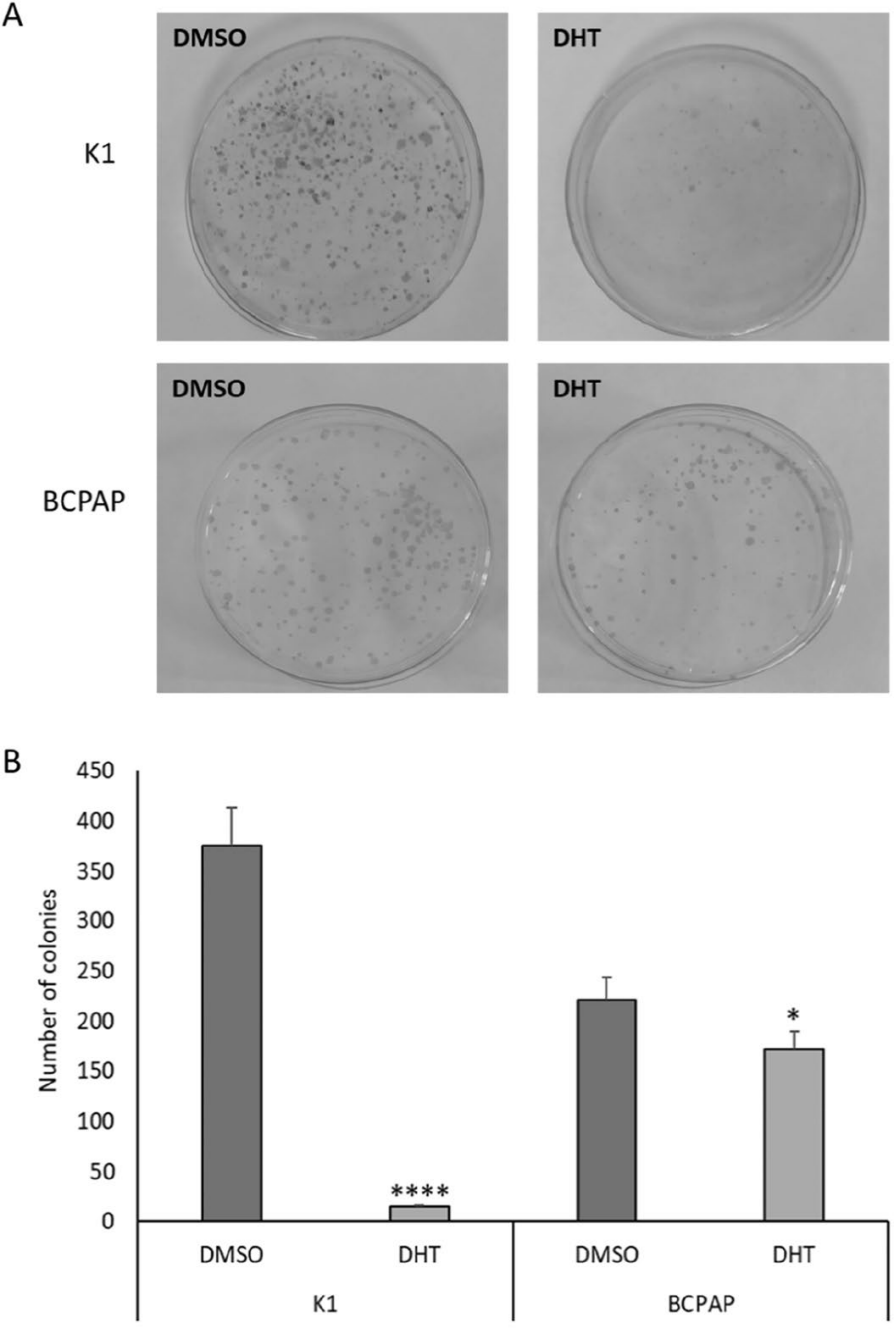
Effects of DHT treatment on colony forming ability in resistant PTC cell lines. The ability to form colonies in an anchorage-dependent way was investigated by colony formation assay in both cell lines following treatment with vehicle (DMSO) and DHT. (**A**) Visual representation of the experiment showing the colonies formed in the plate in both situations (control and treatment) in both cell lines. (**B**) Histograms of the number of colonies per cell line. n = 3; *p < 0.05, ****p < 0.0001.

The effect of this molecule on the ability to form colonies in an anchorage-dependent way in the two cell lines was then investigated. To this aim, we performed a colony formation assay after treatment with vehicle or 2.5 μM DHT. The two cell lines react similarly when treated with DHT. In fact, as shown in Fig. 3, the number of colonies formed after treatment with DHT was strongly reduced in K1 and less markedly also in BCPAP. Despite this slight difference, the reduction was statistically significant in both cell lines.

### Effects of DHT on in vitro tumor cell aggressiveness

**Figure 4:**
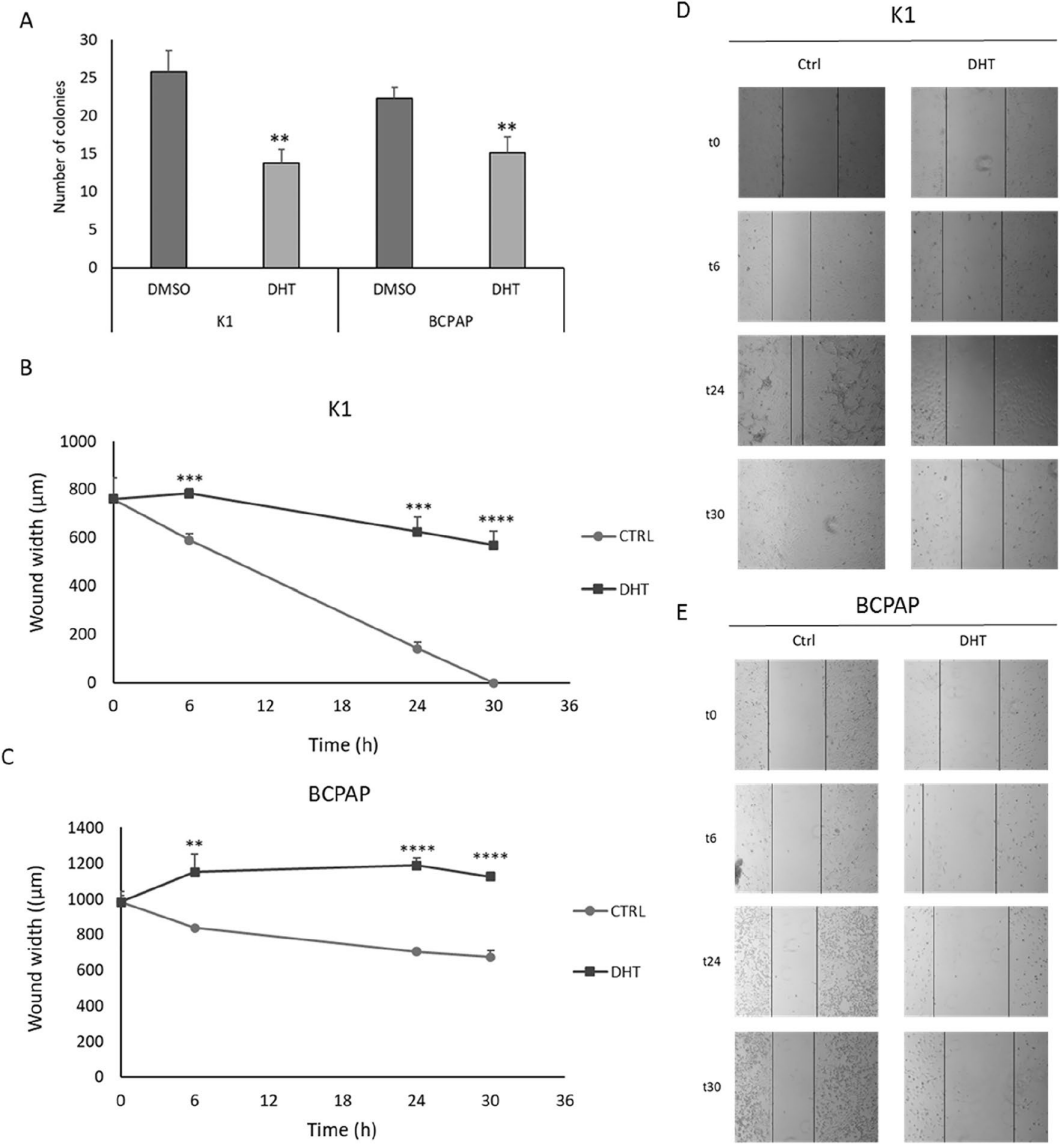
Effects of DHT treatment on the aggressive behaviour of cancer cells. The effect of DHT on the ability to form colonies in an anchorage-independent way was measured by soft agar assay. The data obtained in both cell lines are shown above (**A**). The histograms represent the number of colonies per cell line. Data are representative of 3 independent experiments. (**B**-**E**) Evaluation of the cells ability of to migrate after DHT treatment by wound healing assay. The y-axis shows the width of the wound, given in μm, and the x-axis shows the time, given in hours, in which the measurements were taken. n = 4. **p < 0.01, ***p < 0.001, ****p < 0.0001.

To evaluate the effects of in vitro on PTC cell lines aggressiveness, we first explored the ability of the treated cells to form colonies in an anchorage-independent manner by a soft agar assay, a well- established in vitro method to characterize this competence^29^. As shown in Fig. 4, after treatment with DHT we observed a significant reduction in the number of colonies in both cell lines when compared to DMSO treated cells (panel A). This effect is more evident in K1 cells than in BCPAP cells. In fact, in the first cell line, in the control condition, a mean value of 25 colonies was detected, and this number decreased significantly in the treatment condition (n = 13), thus leading to a reduction of almost 50%. In BCPAP cells, the reduction is lower but still significant: it goes from a mean value of 22 colonies in the control to 15 in the treated cells, therefore with a 33% decrease. Another tumor aggressiveness feature is the migration ability. We performed a scratch assay in order to explore the capacity to migrate of PTC cell lines in the presence or absence of treatment. The 2D wound healing assay is essentially based on the disruption of the cell monolayer at confluence, which generates a cell-free region, which can be re-covered by cells based on their ability to migrate^30^. Figure 4, panels B to E, shows the analysis of the data obtained through this experiment at different time frames (0–30 h). As shown in panels B and D, untreated K1 cells migrated very quickly compared to those treated in which, instead, the migration rate was reduced. Regarding BCPAP cells, in panels C and E, it could be observed that after DHT treatment, the width of the wound does not decrease but increases, the opposite effect than that observed in the control. This finding is probably because in the DHT-treated cells not only a reduced closure capacity was observed, but also cell death that contributed to the enlargement of the wound.

### Effects of DHT on gene expression in PTC cells

**Figure 5:**
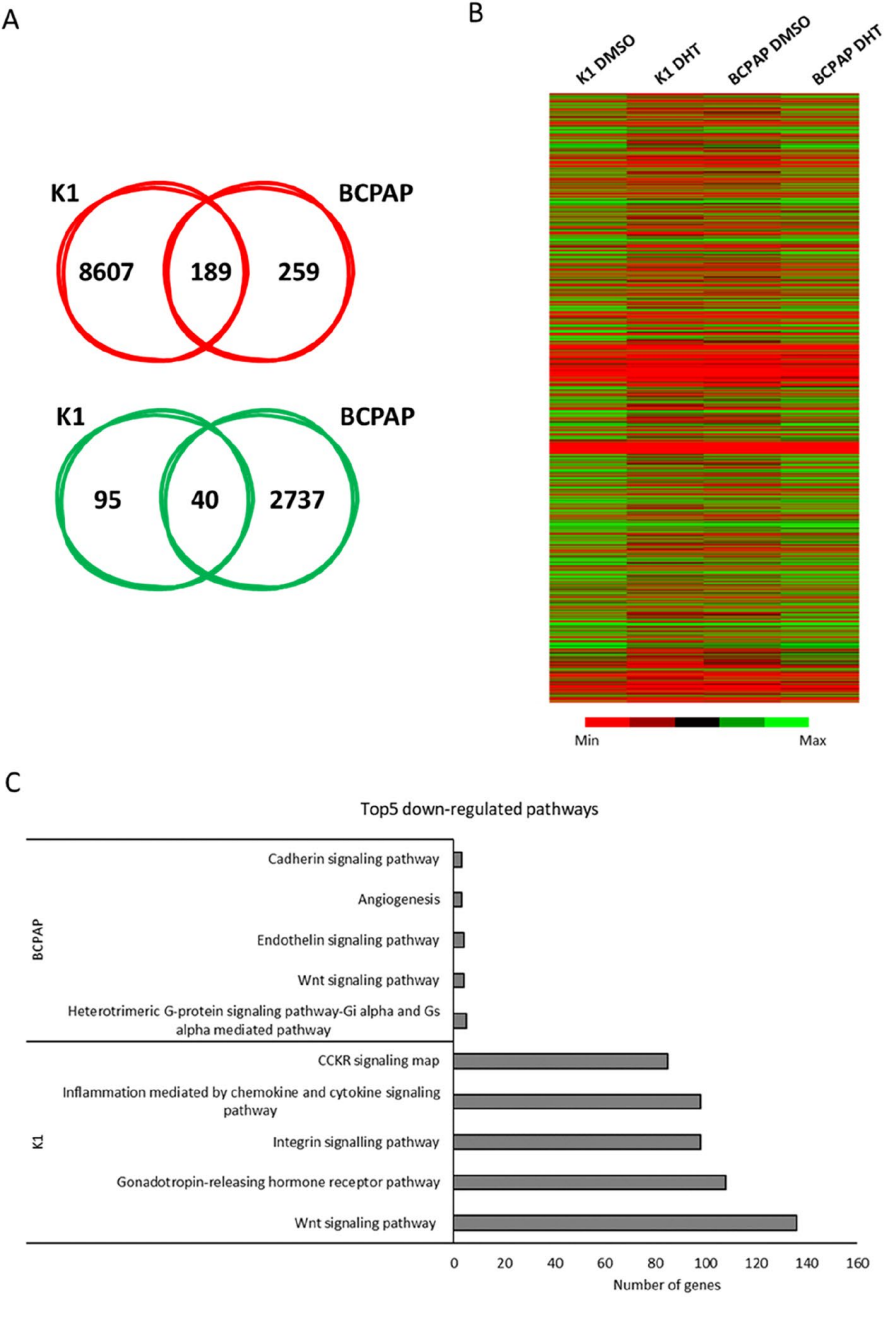
Effects of DHT on gene expression. (**A**) Venn diagrams represented the comparison of downregulated (in red) and upregulated (in green) genes between K1 and BCPAP lines treated with 2.5 μM DHT, after RNA-seq data analysis. Within the intersection of the circles are indicated the alterated genes which are shared between the two cell lines. (**B**) Heat maps obtained after gene expression analysis following treatment with DHT (K1 DHT and BCPAP DHT) or with the vehicle (K1 DMSO and BCPAP DMSO). (**C**) Top5 down-regulated pathways in the two cell lines after DHT treatment, examined independently.

**Figure 6:**
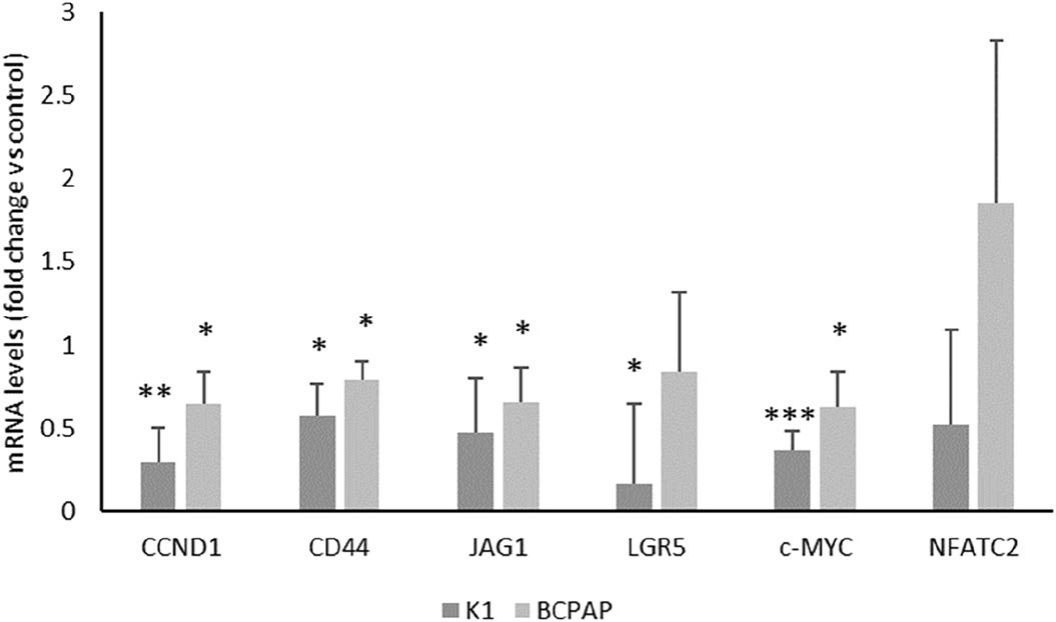
Effects of DHT on genes involved in Wnt pathways. Relative expression levels of *CCND1, CD44, JAG1, LGR5, c-Myc* and *NFATC2* mRNA in K1 and BCPAP cells treated with vehicle (DMSO) or DHT 2.5 μM. For each cell line, mRNA expression in the control cells was set at 1. n = 3. *p < 0.05, **p < 0.001, ***p < 0.0001.

To understand the molecular mechanisms behind the biological effects exhibited by DHT in PTC cell lines, a high-throughput RNA sequencing analysis was performed after 2.5 μM DHT treatment for 6 h. Treatment with DHT resulted in downregulation of 8,607 genes in K1 cells and 259 ones in BCPAP cells. Treatment with DHT induced an upregulation of 95 genes in K1 cells and 2,737 ones in BCPAP cells. Comparing commonly up- and down-regulated genes in the two cell lines, the administration of DHT for 6 h increased the expression of 40 genes and reduced expression of 189 genes (Fig. 5A). Since DHT seems to downregulate a larger number of common gene between K1 and BCPAP than those commonly upregulated, we focused on the commonly downregulated genes. By investigating which pathways were downregulated after DHT treatment in the individual cell lines, we found that the Wnt signaling pathway was the most commonly downregulated (regarding the number of genes involved, 136 in K1 cells and 4 in BCPAP cells) in both cell lines (Fig. 5C). This inspired us to investigate more about the effects of DHT on this pathway. We investigated the levels of expression of known Wnt targets involved in the canonicalpathway (β-catenin-dependent) or the β-catenin-independent (non-canonical) one. After 24 h of treatment the genes *CCND1, CD44, JAG1, LGR5, c-Myc*, involved in the canonical pathway, were significantly reduced (Fig. 6), while *NFATC2* (a non-canonical Wnt pathway actor) did not show significative alterations.

### Effects of DHT on β-catenin protein levels in PTC cells

**Figure 7:**
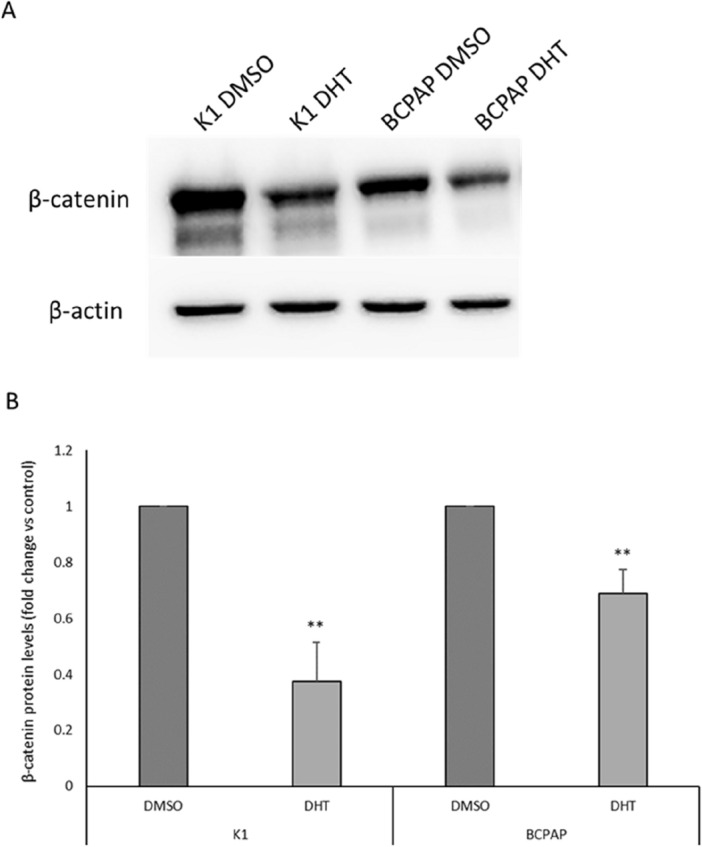
Effects of DHT on β-catenin protein levels. (**A**) Western blot analysis of β-catenin protein level in K1 and BCPAP treated with DHT 5 µM (K1 DHT and BCPAP DHT) or vehicle (K1 DMSO and BCPAP DMSO). (**B**) Densitometric analysis of β-catenin protein levels in K1 and BCPAP cells treated with DHT or vehicle (DMSO). For each cell line, the results were normalized against β-actin levels and expressed as arbitrary unit. n = 3; **p < 0.01.

A final effector after modulation of canonical Wnt signaling pathway is β-catenin^31^.Therefore, we tested whether DHT treatment is able to downregulate β-catenin expression. For this purpose, β-catenin protein levels were evaluated by western blot analysis. As shown in Fig. 7, β-catenin protein levels are significantly reduced in both cell lines after DHT treatment, providing support for the hypothesis that down regulation of the Wnt pathway is a mechanism strongly associated with the effects of DHT administration in these cell lines.

## Discussion

Thyroid cancer is the most common endocrine carcinoma and among its different subtypes the papillary one is the most frequent. Despite PTCs are usually well differentiated, a small percentage of PTCs are characterized by a worse prognosis and more aggressive behavior. Therefore, innovative therapeutic approaches to treat the aggressive PTC fraction are required. In recent years, particular attention has been directed to phytochemical compounds naturally contained in fruits, vegetables, grains, and other plant products. Several studies have demonstrated that these types of compounds are able to influence cell proliferation and cell cycle regulation, and usually are active in multiple signalling pathways that are often disrupted in tumor initiation, proliferation, and propagation. For these reasons these compounds have been widely studied as anticancer drugs for several types of neoplasms, including thyroid cancer, as demonstrated in our previous study on ATC cell lines^26,32^. Among several phytochemicals compounds studied in the literature in this study we focused on 15,16-dihydrotansinone I (DHT), a lipophilic abietane diterpene compound extracted from the dried root of Salvia miltiorrhiza, since it has already shown efficacy in ATC cell lines^26^. We focused on the DHT effects in two PTC-derived cell lines, K1 and BCPAP. In particular, its effect in terms of cell viability, apoptosis, colony formation ability and migration ability was evaluated. Its impact on colony-forming ability in an anchorage-dependent and anchorage-independent manner was investigated with colony formation assay and soft agar assay, respectively. Effects of DHT on colony-forming ability were markedly more evident in the case of K1 leading to minimal values of the number of colonies formed; in BCPAP there was minor, but still significant effect. In addition, the soft agar assay demonstrated that the treatment effect was similar in both cell lines: treatment with DHT 2.5 µM results in a significant decrease in colony number in both cell lines. In particular, we observed a decrease of 50% in K1 cells and about 33% in BCPAP cells. To better investigate in vitro cell aggressiveness cell migration ability was evaluated by using the wound healing assay. It was observed that in relation to the control (vehicle-treated cells), K1 cells show a reduced migratory capacity, even if not zeroed out, as the time they need to close the wound is longer. A different, but at the same time interesting, situation was observed in BCPAP cells. In fact, wound enlargement was observed along time, due to the fact that DHT-treatment inhibits the migratory capacity of cells and causes their death.

Since these preliminary results prove that DHT treatment results in strong biological effects, we decided to proceed with an assessment of gene expression analysis after treatment. Because our goal was to find the primary targets of DHT and minimize secondary effects, an RNA-seq was conducted at early times (6 h). By analyzing the effects of DHT on the expression of down-regulated genes identified after RNA-seq, we found that DHT significantly reduced Wnt signaling pathway in both cell lines (Fig. 4). Wnt signaling pathways include non-canonical and canonical pathways. The canonical pathway involves Wnt and β-catenin, while the non-canonical pathways act independently of β-catenin^33^. The Wnt signaling pathway plays an important role during development and regeneration of adult tissue homeostasis^34^. Abnormal regulation of this pathway is closely associated with several non-cancer diseases and cancer diseases^35^, as also observed by our group^36^, suggesting that the Wnt signaling pathway is an attractive target for disease treatment^37–40^. The canonical Wnt pathway is generally highly conserved and is activated through the binding of extracellular Wnt ligands to membrane receptors by autocrine/paracrine methods. Once activated, the typical Wnt pathway induces the stability of β-catenin which enters the nucleus, and activate expression of genes involved in cell proliferation, survival, differentiation, and migration^37^. In order to confirm the involvement of the canonical Wnt pathway in the response to DHT treatment, the expression of selected genes belonging to the canonical or non-canonical pathway was assessed^41,42^. Gene expression analysis after 24 h of treatment revealed a downregulation of *CCND1, CD44, JAG1, LGR5, c-Myc*, genes involved in the canonical Wnt pathway, while no variations in *NFATC2* mRNA levels were observed. This pattern of gene expression suggests that a significant portion of the effects of DHT is due to the Wnt canonical-pathway alteration.

Considering the importance of β-catenin function and data obtained from RNA-seq, we investigated the effect of DHT on this protein level, since it is the ultimate player in the canonical pathway. Our data clearly indicate that protein expression levels of β-catenin were downregulated, with a greater effect in K1 than in BCPAP, confirming the RNA-seq data. Thus, although specific targets of DHT are still not known, our data indicate that this compound is able to interfere with Wnt/β-catenin signaling pathway. This finding corroborates conclusions of other studies that attribute much of the in vitro antitumor effects of DHT to β-catenin modulation^43,44^.

Overall, though limited to in vitro studies, our results indicate the antitumor properties of DHT on PTC cell lines, suggesting the promising use of this nutraceutical as an adjuvant in the treatment of aggressive papillary thyroid carcinoma.

## Materials and methods

### Cell lines

The human thyroid cancer cell lines derived from PTC used in this study are K1 (Merck KGaA, Darmstadt, Germany) and BCPAP (kindly donated by prof. Russo, University of Catanzaro, Italy). These cell lines had been tested for being mycoplasma-free and Short Tandem Repeat analysis was performed to check the genomic stability of these cell lines. K1 cells were growth in DMEM medium (Euroclone S.p.A, Milano, Italy), while BCPAP cells were growth in RPMI-1640 medium (Euroclone S.p.A). Media were supplemented with 10% FBS (Gibco; Thermo Fisher Scientific, Inc., Waltham, MA, USA), 2 mM L-glutamine (Euroclone S.p.A) and 50 mg/mL gentamicin (Gibco). Cells were cultured in a humidified incubator (5% CO_2_ and 95% air at 37 °C) (Eppendorf AG, Hamburg, Germany). K1 and BCPAP cells were treated with DMSO as vehicle (PanReac AppliChem ITW Reagents, Darmstadt, Germany) and DHT (purity ≥ 99%; Selleck Chemicals, Houston, TX, USA).

### Cell viability

In order to test cell viability, a 3-(4,5-dimethylthiazol-2-yl)-2,5-diphenyltetrazolium bromide (MTT) assay was performed, as previously described^45^. K1 and BCPAP cells were seeded in 96-well plates (4 × 10^3^ cells/well). The following day, cells were treated with DHT (from 0.5 μM to 3 μM) or vehicle (DMSO) at different concentrations. After 24, 48 or 72 h of incubation, 4 mg/mL MTT (Sigma-Aldrich; Merck KGaA) was added to the cell medium and cells were cultured for a further 4 h in the incubator. The supernatant was removed, 100 µL/well DMSO were added, and the absorbance at 570 nm was measured. All experiments were performed as six technical repeats and cell viability is expressed as the fold-change relative to the control (DMSO-treated cells). EC50 was determined from the dose–response curves using the percentage of cell viability.

### Colony formation assay

The clonogenic activity of the PTC cell lines was evaluated by colony formation assay, as previously described^46^. Briefly, K1 and BCPAP were treated with vehicle or DHT and then cells were seeded in 10-cm plates at a density of 2000 and 1000/plate, respectively. Colonies were fixed using methanol and then stained with crystal violet solution, which allows for visual quantification of the number of colonies that expanded.

### Soft agar assay

The clonogenic ability of the K1 and BCPAP cells was studied using a soft agar assay. After 48 h of treatment, cells were collected, and 10,000 cells were suspended in complete medium containing 0.25% agarose (PanReac AppliChem ITW Reagents), then seeded to the top of a 1% agarose complete medium layer in 6-cm plates. The colonies were counted in four different fields, under a Leica DMI-600B inverted microscope (Leica Microsystems Ltd., Wetzlar, Germany).

### Wound healing assay

Papillary thyroid cancer cells’ migratory ability was evaluated by wound healing assay, as previously described ^47^. Briefly, the cells were seeded into six-well plates (150,000 cells/well) and the day after they were treated with DHT or DMSO. A linear scratch was performed with a 200 μL sterile pipette tip across the cell monolayer. Cells were then incubated in a humidified incubator at 37 °C and images of the scratched monolayer were acquired after 0, 6, 24 and 30 h with an inverted microscope Leica DMI-600B (Leica Microsystems Ltd.).

### RNA extraction,high-throughput sequencing and gene expression assay

Total RNA from K1 and BCPAP cells was extracted using RNeasy Mini Kit according to the manufacturer’s instructions (Qiagen, Hilden, Germany). To quantify the RNA through Qubit 4.0 Fluorometer, the Qubit RNA HS assay (ThermoFisher Scientific, Waltham, MA, USA) was used. A total of 1 μg total RNA from all cell lines was extracted as described above and reverse transcribed to cDNA was generated using the SuperScript IV VILO Kit (ThermoFisher Scientific). Barcoded libraries were prepared using the Ion AmpliSeq Transcriptome Panel Human Gene Expression CORE (Thermo Fisher Scientific, Waltham, MA, USA) and the Ion AmpliSeq Library Kit Plus (Thermo Fisher Scientific), following the manufacturer’s protocol. Veriti Dx 96-Well Thermal Cycler (Applied Biosystems, Waltham, MA, USA) was used for all reactions. Barcoded libraries were quantified with the Qubit dsDNA HS Assay kit (Life Technologies, Carlsbad, CA, USA) and then diluted to 100 pM. Libraries were loaded into the Ion Chef instrument (Thermo Fisher Scientific) for template enrichment and chip loading. Sequencing was performed with the Ion S5 GeneStudio Sequencer using the Ion 540 Kit-Chef and the Ion 540 chip-kit (all Thermo Fisher Scientific). Reads were aligned to the reference genome and the RNA-seq analysis plugin was run on the Torrent Suite Server (Thermo Fisher Scientific). As previously described^36^, for further analysis, we selected effective data for those with FPKM values > 0.5 and log2 fold-change > 1.5 or <  − 1.5. In the evaluation of commonly up or downregulated genes, the cut-offs were increased to − 3 and + 3 in order to obtain more representative results. The heatmap was created by processing the sequencing data with the Morpheus online tool (https://software.broadinstitute.org/morpheus).

To evaluate the gene expression of selected genes, 500ng of total RNA were reverse transcribed to cDNA using random exaprimers and SuperScript III reverse transcriptase (Life Technologies, Carlsbad, CA, USA). Real-time PCRs were performed using Platinum Sybr Green QPCR supermix (Life Technologies) with the QauntStudio3 Systems (Applied Biosystems). The ∆∆CT method, by means of the SDS software (Applied Biosystems), was used to calculate mRNA levels. Oligonucleotide primers were purchased from Sigma Aldrich and the sequences are available upon request.

### Protein extraction and western blot

K1 and BCPAP cells were collected by scraping and lysed with total lysis buffer (Tris HCl 50 mM pH8, NaCl 120 mM, EDTA 5 mM, Triton 1%, NP40 1%, protease inhibitors), as previously described^32^. Lysates were centrifuged at 13,000×*g* for 10 min at 4 °C, and supernatants were quantified using the Bradford assay. For western blot analysis, the same protocol previously described here was used^48^. Briefly, proteins were electrophoresed on 10% SDS-PAGE and then transferred to nitrocellulose membranes, saturated with 5% non-fat dry milk in PBS/0.1% Tween 20. To evaluate PARP, c-PARP and β-catenin protein levels, 60 ng protein were electrophoresed on 7.5% SDS-PAGE and then transferred to nitrocellulose membranes, saturated with 5% non-fat dry milk in PBS/0.1% Tween 20. Blots have been cut prior to hybridization with antibodies. The membranes were then incubated overnight with rabbit monoclonal anti-PARP antibody 1:1000 (Abcam, Cambridge, UK), or mouse monoclonal anti-β-catenin antibody 1:1000 (Santa Cruz Biotechnology), or rabbit anti-β-actin antibody 1:2000 (Abcam, Cambridge, UK). The following day, membranes were incubated for 2 h with peroxidase-conjugated anti-mouse or anti-rabbit IgG secondary antibody, respectively (Sigma-Aldrich, St. Louis, MO, USA). Blots were developed using UVITEC Alliance LD (UVITec Limited, Cambridge, UK) with SuperSignal Technology (Thermo Fisher Scientific).

### Statistical analysis

All experiments were performed in triplicate and data obtained are expressed as means ± standard deviation. Significances were analyzed with the student's t-test performed with GraphPAD Software for Science (San Diego, CA, USA).

### Supplementary Information

Supplementary Information.

Supplementary Table S1.

## Data Availability

The datasets generated and/or analyzed during the current study are available from the corresponding author on reasonable request.
