# Supplementary figures and images for: Dihydrotanshinone I exhibits antitumor effects via β-catenin downregulation in papillary thyroid cancer cell lines
Source: Sci Rep. 2024 Apr 3;14:7853. doi: 10.1038/s41598-024-58495-9 (PMC10991365; doi:10.1038/s41598-024-58495-9)

Original blots for Figure 2A

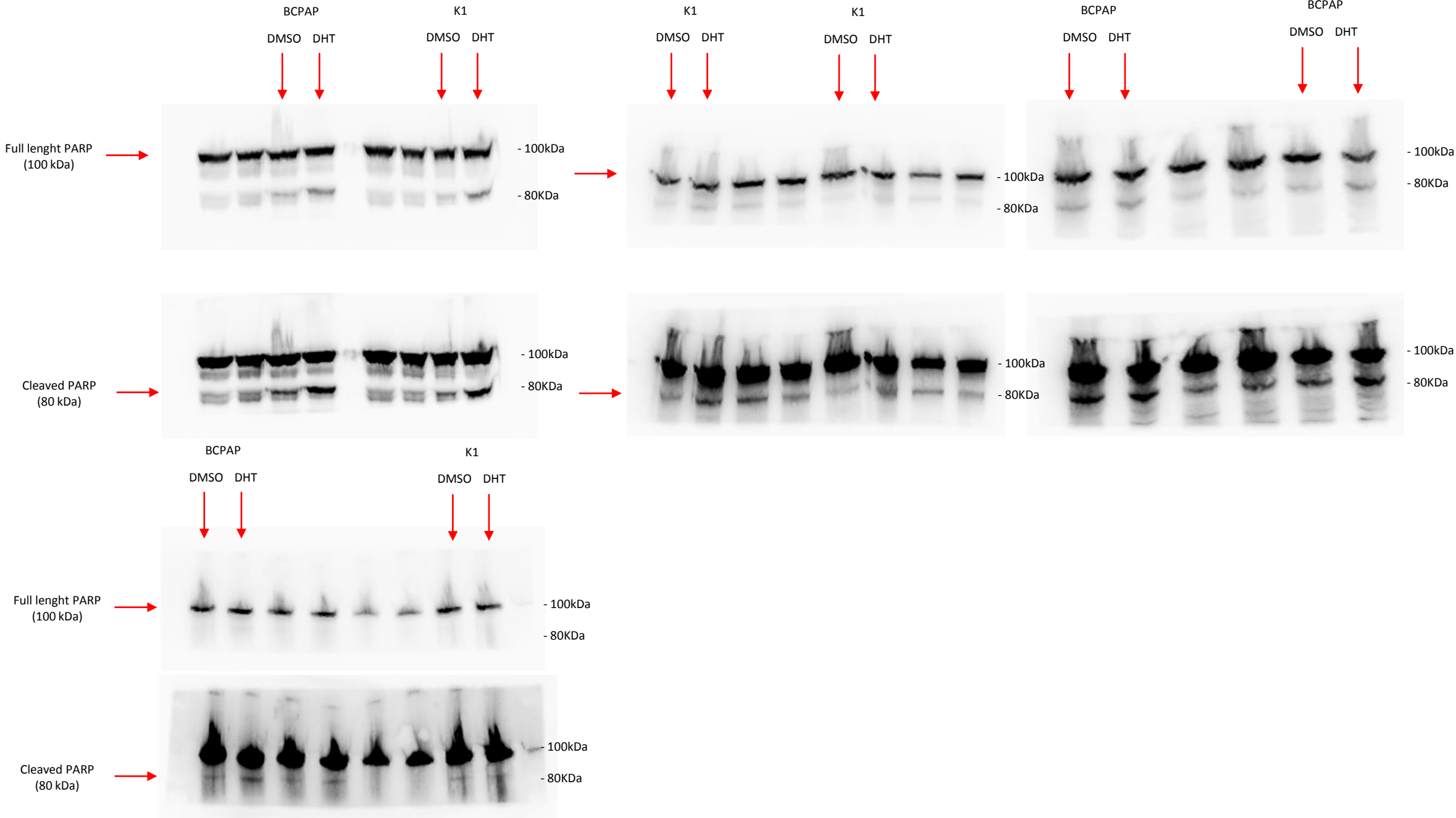

Original blots for Figure 7A

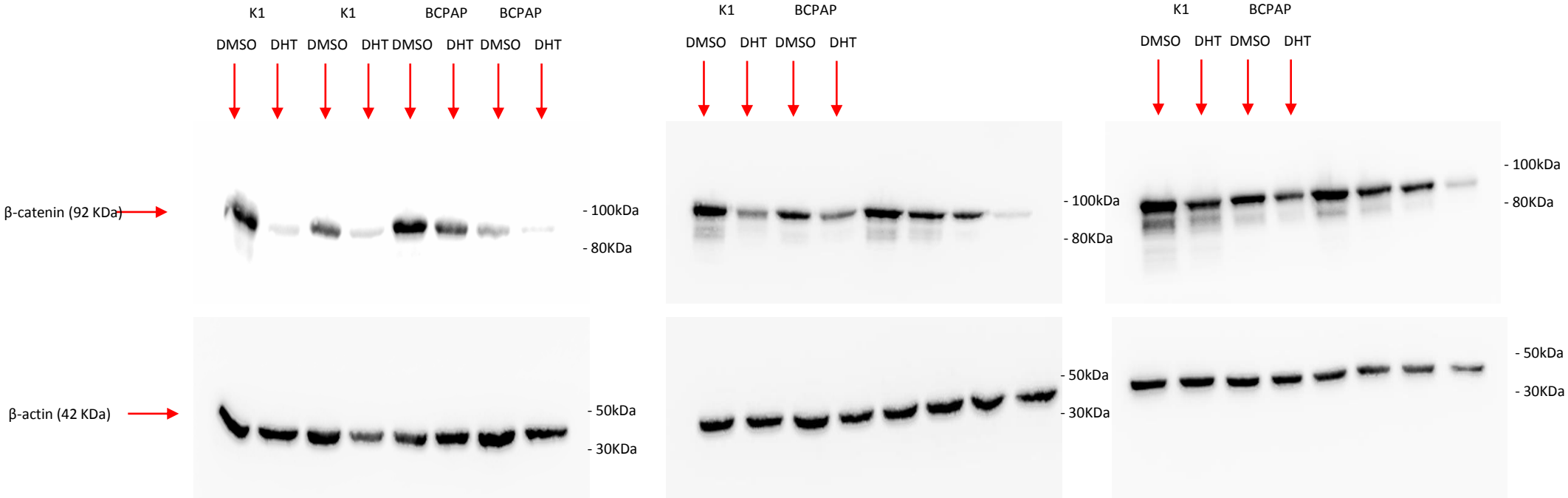

Supplement: Supplementary file 1 — Supplementary Information. [file 41598_2024_58495_MOESM1_ESM.pdf]
