# Supplementary material for: Dihydrotanshinone I exhibits antitumor effects via β-catenin downregulation in papillary thyroid cancer cell lines
Source: Sci Rep. 2024 Apr 3;14:7853. doi: 10.1038/s41598-024-58495-9 (PMC10991365; doi:10.1038/s41598-024-58495-9)
Supplement: Supplementary file 2 — Supplementary Table S1. [file 41598_2024_58495_MOESM2_ESM.pdf]

Dihidrotanshinone I exhibits antitumor effects via β-catenin downregulation in papillary thyroid cancer cell lines, by Elisabetta Molteni, Federica Baldan, Giuseppe Damante and Lorenzo Allegri.

		24h	*48h*	*72h*
K1	0.5 µM	**	**	**
	1 µM	***	****	***
	1.5 µM	**	****	****
	3 µM	****	****	****
BCPAP	0.5 µM	****	****	***
	1 µM	***	****	****
	1.5 µM	****	****	****
	3 µM	****	****	****

Supplementary Table S1. p-value of the MTT assay with DHT. p-values obtained in the MTT assay with different doses of DHT (from 0.5 µM to 3 µM) compared with the control treatment (DMSO) are enlisted here. ** $p < 0.01$, *** $p < 0.001$, **** $p < 0.0001$
